# Male reproductive aging arises via multifaceted mating-dependent sperm and seminal proteome declines, but is postponable in *Drosophila*

**DOI:** 10.1073/pnas.2009053117

**Published:** 2020-07-01

**Authors:** Irem Sepil, Ben R. Hopkins, Rebecca Dean, Eleanor Bath, Solomon Friedman, Ben Swanson, Harrison J. Ostridge, Lucy Harper, Norene A. Buehner, Mariana F. Wolfner, Rebecca Konietzny, Marie-Laëtitia Thézénas, Elizabeth Sandham, Philip D. Charles, Roman Fischer, Josefa Steinhauer, Benedikt M. Kessler, Stuart Wigby

**Affiliations:** ^a^Department of Zoology, University of Oxford, OX1 3SZ Oxford, United Kingdom;; ^b^Department of Ecology and Evolution, University of California, Davis, CA 95616;; ^c^Department of Genetics, Evolution and Environment, University College London, WC1E 6BT London, United Kingdom;; ^d^Department of Biology, Yeshiva University, New York, NY 10033;; ^e^School of Biology, University of St Andrews, KY16 9ST St Andrews, United Kingdom;; ^f^Department of Molecular Biology and Genetics, Cornell University, Ithaca, NY 14853;; ^g^Nuffield Department of Medicine, TDI Mass Spectrometry Laboratory, Target Discovery Institute, University of Oxford, OX3 7FZ Oxford, United Kingdom;; ^h^Faculty Biology, Applied Zoology, Technische Universität Dresden, 01069 Dresden, Germany;; ^i^Institute of Infection, Veterinary and Ecological Sciences, University of Liverpool, L69 7ZB Liverpool, United Kingdom

**Keywords:** seminal fluid, sperm, aging, fertility, fitness

## Abstract

Declining ejaculate performance with male age is taxonomically widespread and has broad fitness consequences. Ejaculate success requires fully functional germline (sperm) and soma (seminal fluid) components. However, some aging theories predict that resources should be preferentially diverted to the germline at the expense of the soma, suggesting differential impacts of aging on sperm and seminal fluid and trade-offs between them or, more broadly, between reproduction and lifespan. While harmful effects of male age on sperm are well known, we do not know how much seminal fluid deteriorates in comparison. Moreover, given the predicted trade-offs, it remains unclear whether systemic lifespan-extending interventions could ameliorate the declining performance of the ejaculate as a whole. Here, we address these problems using *Drosophila melanogaster.* We demonstrate that seminal fluid deterioration contributes to male reproductive decline via mating-dependent mechanisms that include posttranslational modifications to seminal proteins and altered seminal proteome composition and transfer. Additionally, we find that sperm production declines chronologically with age, invariant to mating activity such that older multiply mated males become infertile principally via reduced sperm transfer and viability. Our data, therefore, support the idea that both germline and soma components of the ejaculate contribute to male reproductive aging but reveal a mismatch in their aging patterns. Our data do not generally support the idea that the germline is prioritized over soma, at least, within the ejaculate. Moreover, we find that lifespan-extending systemic down-regulation of insulin signaling results in improved late-life ejaculate performance, indicating simultaneous amelioration of both somatic and reproductive aging.

## Significance

Reduced fertility with advancing age is well known in females but understudied in males. Most previous work on male reproductive aging has focused on age-related effects on sperm. However, nonsperm seminal fluid is also vital for fertility but might age differently. Using fruit flies, we find that seminal fluid and sperm are both qualitatively and quantitatively affected by age with each making distinct contributions to declining reproductive performance in older males. However, the relative impacts on sperm and seminal fluid often differ, leading to mismatches between ejaculate components. Despite these differences, experimental extension of male lifespan can improve overall ejaculate performance in later life, suggesting that such interventions can delay both male reproductive aging and death.

Research on a wide range of animal taxa provides accumulating evidence that increased male age reduces ejaculate performance (1, 2). From an evolutionary perspective, a loss of fertility with age has fitness impacts not just on the male, but also on his mates, thereby shaping sexual selection and sexual conflict (2). Most prior work in this area has focused on the impact of male age on the germline component of the ejaculate: sperm. Declines in sperm count, motility, and viability with age are common but not ubiquitous (reviewed in ref. 3). Harmful effects on sperm present an obvious cost to males and to any mates of old males that lack alternative fertilization opportunities (2). Moreover, in species where females mate with multiple males, such that the sperm of multiple males compete for fertilization ("sperm competition") (4), impaired sperm performance is likely to be especially harmful to males, because rivals with healthy sperm might monopolize fertilizations (5).

But ejaculates contain much more than sperm, and ejaculate functions extend beyond fertility. Ejaculates also contain nongermline seminal fluid, which is a complex mixture of molecules that makes vital contributions to ejaculate function (6). In addition to supporting sperm and promoting fertility and sperm competitiveness, seminal fluid can alter female physiology and in some taxa change female behavior, enhancing the male’s competitive reproductive success and even influencing offspring health (7–9). The impact of male age on the ejaculate, therefore, has the potential to influence male reproductive success via multiple pathways, through alterations to the seminal fluid and its functions. Indeed, there is some existing evidence to support this. A recent study has shown associations between distinct seminal proteome profiles and sperm speed in aging red junglefowl males (10). In *D. melanogaster*, males exhibit age-related changes in the expression of some seminal fluid protein (Sfp) genes (11), and increased male age reduces fertility and other aspects of ejaculate performance, such as sperm competitiveness and inhibition of female sexual receptivity (11–13). However, whether these age-related seminal proteome changes translate into reduced ejaculate performance remains to be tested, and its importance relative to changes to sperm remains unknown.

Classical aging theory postulates a resource trade-off broadly between reproduction and survival. Moreover, the disposable soma theory specifically predicts that resources are prioritized for germline maintenance over the soma (14). The ejaculate consists of both germline (sperm) and soma (seminal fluid) meaning that there is the potential for trade-offs between ejaculate components and/or differential impacts of male age on sperm and seminal fluid. Furthermore, mechanisms promoting somatic maintenance for improved lifespan might come at the expense of the germline (i.e., sperm) or, more generally, suppress reproductive tissues (i.e., the whole ejaculate). Evidence in some species supports the idea of a trade-off between sperm production and lifespan (15–17) or between the ejaculate and the other key life-history traits, such as immunity (18), although some evidence suggests otherwise (19). Consistent with the idea that lifespan-extending manipulations in males act at the expense of fertility, one mouse study found that rapamycin treatment led to increased survival but concomitant testicular shrinkage (20). However, we do not understand the relative impacts of male age on the varied components of the ejaculate, both germline (sperm) and somatic (seminal fluid), and crucially how these changes link to fertility, sperm competitiveness, and other aspects of ejaculate function, such as stimulating postmating changes in females. For example, is sperm production generally prioritized over seminal fluid, potentially leading to mismatches in their aging patterns? Does delayed death come with an obligatory cost to the ejaculate? Despite a revolution in aging research over the past two decades, which has identified key evolutionarily conserved lifespan-mediating genetic pathways (21), our understanding of the mechanisms mediating reproductive senescence in males, and potential trade-offs between the ejaculate and lifespan remains limited.

Here, we use *Drosophila* to first dissect the contributions of age-related changes to sperm and Sfps to a suite of key ejaculate functions (e.g., fertility, fecundity, sperm competitiveness, and female refractoriness, refs. 8, 22). Previous work indicates that sexual activity can profoundly shape patterns of male reproductive aging in this species (23), but the potential interacting impacts of mating and aging on sperm and Sfps—their production and replenishment—and the reproductive consequences of each, have not been fully explored. We, therefore, examine the impacts of aging in both sexually active and sexually inactive males. Using a mix of proteomic, cell-labeling, and biochemical approaches, we specifically test the idea that the cumulative effects of aging and mating impact the abundance and quality of Sfps as well as sperm, and that these effects on Sfps and sperm contribute to distinct aspects of declining ejaculate performance. By assessing the relative role of Sfps and sperm in male reproductive aging, we test the idea that the germline should receive resource priority. Finally, we investigate the impact of a somatic lifespan-extending intervention on ejaculate performance to explore whether the manipulation of the conserved insulin signaling pathway trades off against overall ejaculate performance or, alternatively, enhances it in late life (24).

## Results and Discussion

### Reproductive Consequences of Male Aging and Mating History.

**Fig. 1.**
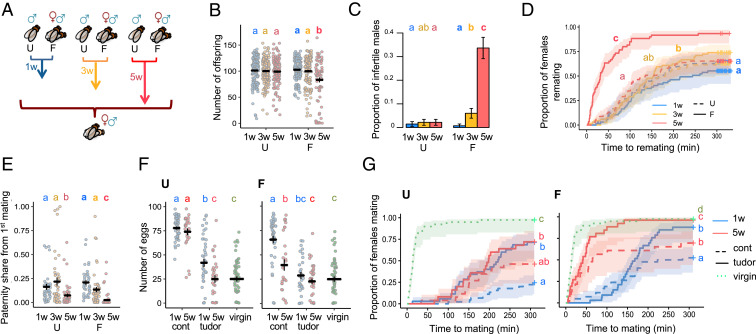
Decline in reproductive performance in response to male age and mating activity. (*A*) Experimental design. U and F males. (*B*) Number of offspring from a single mating, excluding infertile matings (age and mating interaction: χ^2^_2_ = 98.668; *P* = 0.0005) (*n* = 73–135). (*C*) The proportion of infertile matings (age and mating interaction: χ^2^_2_ = 11.32; *P* = 0.0035) (*n* = 110–137). (*D*) Female latency to remate (age and mating interaction: χ^2^_2_ = 34.886; *P* < 0.0001) (*n* = 60–70). (*E*) Paternity share of the experimental first male (age and mating interaction: χ^2^_2_ = 219.34; *P* = 0.0234) (*n* = 37–55). (*F*) Number of eggs laid by virgin females or females mated to spermless (tudor) or control (cont) experimental males (*U:* female mating treatment: χ^2^_4_ = 2398.7; *P* < 0.0001; *n* = 29–56) (*F:* female mating treatment: χ^2^_4_ = 1405.9; *P* < 0.0001; *n* = 29–56) (*G*) Female latency to mate for virgin females and females first mated to spermless (tudor) or control (cont) experimental males (*U:* female mating treatment: χ^2^_4_ = 137.923; *P* < 0.0001; *n* = 25–45) (*F:* female mating treatment: χ^2^_4_ = 99.421; *P* < 0.0001; *n* = 23–42). Differences at *P* < 0.05 within mating groups and age categories and between treatments are represented as different letters.

To examine the contributions of sperm and seminal fluid deterioration to male reproductive aging, we began by measuring traits known to be mediated by sperm and Sfps (8). We measured reproductive traits in experimental males that were 1-wk-old (1w, young), 3-wk-old (3w, middle aged), or 5-wk-old (5w, old) and had been maintained in either single-sex groups of 12 (unmated [U]) or in groups of three males and nine females (frequently mated [F]) (Fig. 1*A*). These time points span male peak reproductive performance (1w, young) and reproductively senesced states (5w, old) while ensuring that most males survive the experiment (13) (*SI Appendix*, Fig. S1). The two mating treatments (U or F) deliberately represent two extremes, abstinent versus fully sexually active, designed to expose the full potential range of male reproductive aging processes that might occur in varying mating environments.

As expected, based on previous work (12, 23, 25), we found clear evidence that male reproductive function declines with age. However, the effects are highly dependent on male sexual activity. Old-F males father fewer offspring, are more likely to be infertile, are poorer at suppressing female remating, and their sperm perform poorly when competing with the ejaculates of rival males (Fig. 1 *B*–*E*). Old-U males are also poor sperm competitors, but their reproductive output, fertility, and ability to suppress female remating are not significantly reduced compared to young males (Fig. 1 *B*–*E*). Old males also show a significant reduction in copulation probability and an increase in mating latency—an effect again exacerbated by frequent mating (*SI Appendix*, Fig. S2)—but show a significant increase in mating duration in the U group (*SI Appendix*, Fig. S3). A similar increase in mating latency with age has been recently shown for U males but not for U females (26).

We also measured the impact of male age and mating history on female egg laying in wild-type males. We found that Old-F males are poorer at stimulating female egg laying such that Old-F mated females have significantly lower fecundity relative to all other mated treatments, and these eggs are less likely to hatch (*SI Appendix*, Fig. S4). However, male age and mating history do not have a significant effect on the viability of hatched eggs developing to pupae or adulthood (*SI Appendix*, Fig. S5).

Taken together, our results highlight how frequent mating is an important contributor to age-dependent reproductive decline in males. Sperm and seminal fluid are the prime candidates for mediating the age-related decline in postmating phenotypes (reproductive output, fertility, ability to suppress female remating, and sperm competitiveness), given their known essential roles (27). However, nonejaculate effects of male age on female traits (e.g., pheromonal, which can vary with age and mediate attractiveness in other insects, ref. 28) cannot be completely excluded.

Next, we investigated whether the seminal fluid alone, in the absence of sperm, contributes to age-related reproductive decline. Long-term elevated egg production and sexual refractoriness in females require the receipt of sperm as well as Sfps, but these responses can also be partially elevated in the short-term without sperm (29). We mated females to either spermless (*son-of-tudor*) males or control males (which transfer both sperm and seminal fluid) of varying age and mating environment or kept the females as virgins to examine nonsperm effects of male age and mating on female fecundity and sexual refractoriness. We first found that both age and mating activity impact the ability of spermless males to induce egg laying in their mates. Old-U, Old-F, and Young-F spermless males are all poor at stimulating female fecundity relative to Young-U spermless males (Fig. 1*F*). Among the sperm-producing controls, which, as expected, induced higher levels of fecundity overall than spermless males, only the mates of Old-F males had significantly lower fecundity relative to young males. Old-U control males were not significantly worse at fecundity stimulation than Young-U males (Fig. 1*F*), confirming the patterns we have seen previously in wild-type flies (*SI Appendix*, Fig. S4).

Next, we found that male age also impacts the ability of spermless males to suppress female remating, but only in the F treatment (Fig. 1*G*). Old-F spermless males are poorer at suppressing female remating than Young-F spermless males, but both still inhibit female receptivity relative to virgins. As expected, F controls perform better than F spermless males overall, but there is still a significant impact of age. Among the U males, all induce a higher degree of refractoriness in females relative to virgins, but age has no significant effect on the performance of spermless males. U controls perform generally better than U spermless males, but not significantly so for Old-U males (Fig. 1*G*).

The ejaculates of *son-of-tudor* males do not contain sperm (30), meaning that the age-related declines in reproductive function seen in these experiments are very likely due to changes in Sfps; some of these proteins are known to be necessary and sufficient for stimulating fecundity and refractoriness responses in females (8). Our experiments, therefore, expose the potent but previously unrecognized impact of age on seminal fluid performance, which would otherwise be masked by sperm effects in wild-type males.

### Quantitative Age and Mating Effects on the Seminal Proteome.

Having identified loss of ejaculate performance associated with increased age and mating activity, and the direct contribution of the seminal fluid, we next investigated whether changes to the seminal fluid proteome could explain these effects. We applied label-free quantitative proteome analysis to the Sfp-producing tissues (accessory glands and ejaculatory duct) of experimental males (31–33).

**Fig. 2.**
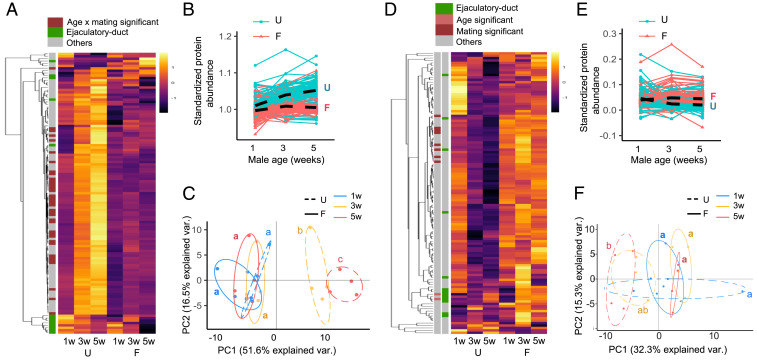
The seminal fluid proteome responds differentially to aging in U versus F males. (*A*) Heatmap of the abundance of the 117 Sfps detected in accessory gland and ejaculatory duct samples (*n* = 4 replicate experiments per group). The abundance of 40 out of 117 Sfps exhibit a significant differential response to age and mating after multiple test corrections. The annotation classification of each Sfp is indicated. (*B*) Line plots showing the change in standardized Sfp abundance with age. The average change in Sfp abundance for U and F males is depicted with lines marked U and F, respectively (age and mating interaction: L ratio^2^_2_ = 163.856; *P* < 0.0001). (*C*) Principal component analyses of the seminal fluid proteome in male reproductive tissues (age and mating interaction: L ratio^2^_2_ = 34.949; *P* < 0.0001). (*D*) Heatmap of the abundance of 117 seminal fluid proteins transferred to females during mating. None of the individual 117 Sfps exhibited a significant interaction between age and mating group after multiple test corrections. Two ejaculatory duct-specific Sfps were transferred in significantly higher quantities in response to age, independent of mating activity (CG17242 and CG5162), and 10 Sfps were transferred in significantly higher quantities in response to frequent mating independent of age (Acp26Aa, CG10587, CG17472, CG3097, CG34002, Est-6, NLaz, Regucalcin, Sfp24F, and Sfp65A). The annotation classification of each Sfp is indicated. (*E*) Line plots showing the standardized abundance of Sfps transferred with age. The average change in Sfp abundance for U and F males is depicted with lines marked U and F, respectively (age and mating interaction: L ratio^2^_2_ = 130.595; *P* < 0.0001). (*F*) Principal component analyses of the seminal fluid proteome transferred to females (age and mating interaction: L ratio^2^_2_ = 11.485; *P* = 0.003). Differences at *P* < 0.05 within mating groups and age categories are represented as different letters.

Focusing our analyses on established Sfps (31, 34) and examining first the production of Sfps by males before transfer to females, we found that the abundance of 40 out of 117 Sfps exhibited a significant differential response to age and sexual activity (Fig. 2 *A* and *B*). Many of the Sfps showed an increase in Old-U males, mirrored by an increase in the size of the accessory gland, the tissue that makes most Sfps (*SI Appendix*, Fig. S6), but these changes were absent in Old-F males. A principal component analysis supported these findings, showing that the composition of the seminal fluid proteome changes significantly with age in U males but not in F males (Fig. 2*C*). All of the up-regulated Sfps are specific to the accessory glands (31) and include Sfps that function in sperm storage and female postmating behavior modification (8) (*SI Appendix*, Table S1). In contrast, the abundance of most of the ejaculatory duct derived Sfps (31, 35) do not exhibit a differential response to age and mating, and cluster separately from the rest of the proteins (Fig. 2*A* and *SI Appendix*, Table S2). Notably, this disparity indicates that the two male reproductive tissues respond differentially to age and mating. Previous work has revealed that accessory gland secondary cells grow preferentially compared to main cells with age and mating (36). Our results suggest a further age-related mismatch in secretory activity between the accessory glands and the ejaculatory duct.

By comparing the quantity of Sfps present in males before and after mating, we can infer Sfp transfer to females during copulation (31, 32). Transferred Sfps show an age-related decline in U males (Fig. 2 *D* and *E*), despite their higher accumulation in the accessory glands (Fig. 2 *A*–*C*): i.e., despite producing Sfps in greater abundance, Old-U males appear to be poor at transferring them to females during copulation. A principal component analysis further shows that the composition of the seminal fluid proteome transferred changes significantly with age in U males but not in F males (Fig. 2*F*). We again observed separate clustering for several ejaculatory duct specific Sfps where the trend is an increase in Sfp transfer with age in F males (Fig. 2*D*), although this effect was weaker than the differences seen in Sfp production (Fig. 2*A*).

### Qualitative Age and Mating Effects on the Seminal Proteome.

**Fig. 3.**
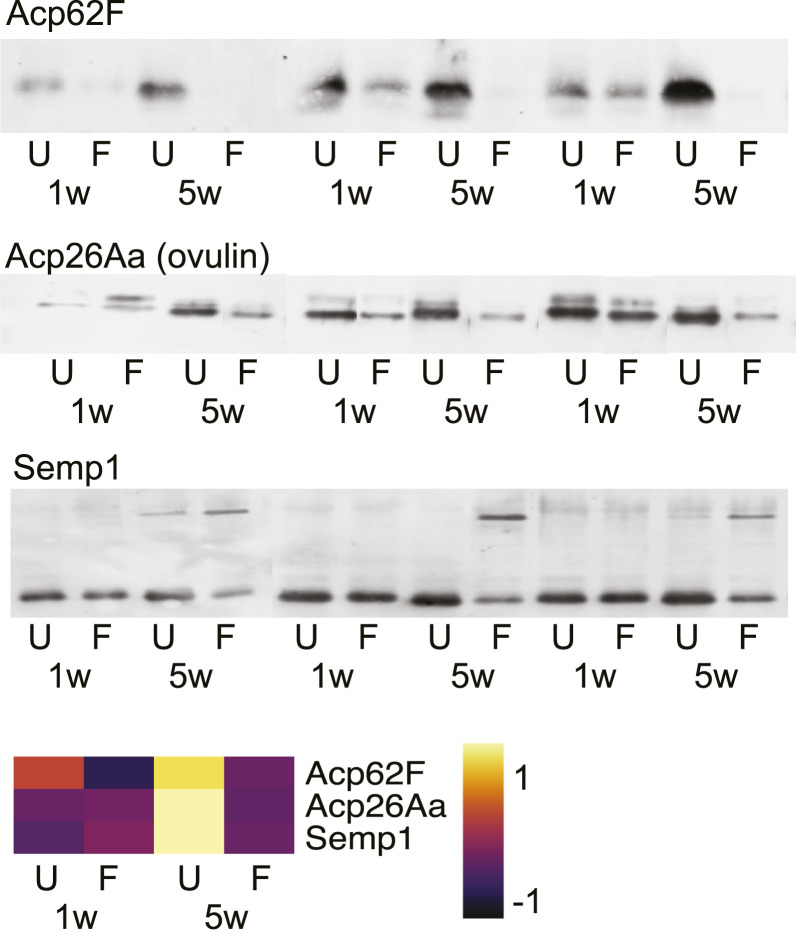
The gel mobility of a number of functionally important Sfps (Acp62F, Acp26Aa, and Semp1) as determined by Western blots, in 1w and 5w males from U and F groups. The abundance of each protein is predicted from the proteomic data and illustrated as a heatmap. Each lane is an individual male. *n* = 3–6. Full blots are shown in *SI Appendix*, Fig. S7.

Next, we tested for protein quality changes by performing Western blot analyses on a subset of six functionally important Sfps: Acp62F, Acp26Aa (ovulin), Semp1, Acp65DE, Acp70A (sex peptide), and CG9997. For each Sfp tested, our proteomic data, which are based on trypsin-cleaved peptides, indicate either an age-related increase in U males or no change in F males in Sfp abundances (Fig. 3 and *SI Appendix*, Fig. S7). Surprisingly, therefore, Acp62F is largely undetectable by Western blot in Old-F males (Fig. 3 and *SI Appendix*, Fig. S7). This shows that aging degrades Acp62F—an Sfp that has previously been implicated in sperm competition (37)—in such a way that, while no band is detectable on Western blot, the trypsin-cleaved peptides remain identifiable by mass spectrometry.

The double band of Acp26Aa (ovulin), one of the most rapidly evolving proteins in *Drosophila* (38), becomes either more condensed or loses the top band altogether in old males, representing possible age-specific alternative splicing or posttranslational modification. Likewise, Semp1, a seminal metalloprotease that cleaves ovulin within females (39), shows an additional upper band in old males from both mating groups, indicating a potential large age-specific posttranslational modification or attachment to a larger protein (Fig. 3 and *SI Appendix*, Fig. S7). Full-length ovulin and two of its cleavage products stimulate ovulation (40), hence, any deterioration in it, or in interacting proteins, such as Semp1, has the potential to negatively impact female ovulation rate following mating (Fig. 1*F*). We saw no qualitative changes in the three other Sfps tested (Acp70A [sex peptide], Acp36DE, and CG9997; *SI Appendix*, Fig. S7). Together, these results indicate that a subset of seminal proteins display qualitative alterations in response to age and, in some cases the combination of age and frequent mating.

Taking our proteomics and Western blot data together, our results suggest that a distinct set of Sfps accumulates with age in male reproductive tissues in the absence of mating, resulting in compositional change in the seminal proteome (Fig. 2 *C* and *F*). The results also suggest seminal proteome imbalance and suboptimal transfer, contribute to the age-related sperm competitiveness decline in sexually abstinent males (Fig. 1*E*) and reduced fecundity stimulation in spermless males (Fig. 1*F*) perhaps due to harmful accumulation within the accessory glands that prevents normal ejaculation. In contrast, the abundance and transfer of Sfps is maintained with age in the presence of frequent mating, which, given the striking decline in many measures of ejaculate performance in Old-F males, indicates that quantitative Sfp effects provide, at most, a negligible contribution to male age-related ejaculate deterioration in these sexually active males. Instead, our data are more consistent with qualitative changes to the seminal proteome being the main cause of nonsperm ejaculate performance loss with male age in sexually active males.

### Seminal Fluid Decline Does Not Extend to Sperm-Protecting Function.

The qualitative changes we detect are consistent with a loss of seminal protein homeostasis (41) and are likely to contribute to the compromised postmating phenotypes observed in females mating with old males. A previous study using fluorescently stained sperm suggested that seminal fluid improves sperm survival, even if the sperm are from a different male (42). We, therefore, tested whether the age-related qualitative changes in seminal fluid were associated with a reduction in the sperm-protecting function of seminal fluid. Using the same methods as previous work (42)—SYBR-14 and propidium iodide fluorescent staining—to stain live and dead sperm cells, respectively, we measured the effects of seminal fluid on the survival of sperm recovered from a different male. However, we found no evidence that age or mating history compromises the ability of seminal fluid to keep sperm alive, and in contrast to previous work (42), no evidence to support the idea that seminal fluid from a different male protects sperm (*SI Appendix*, Fig. S8).

### Age and Mating Effects on Sperm.

**Fig. 4.**
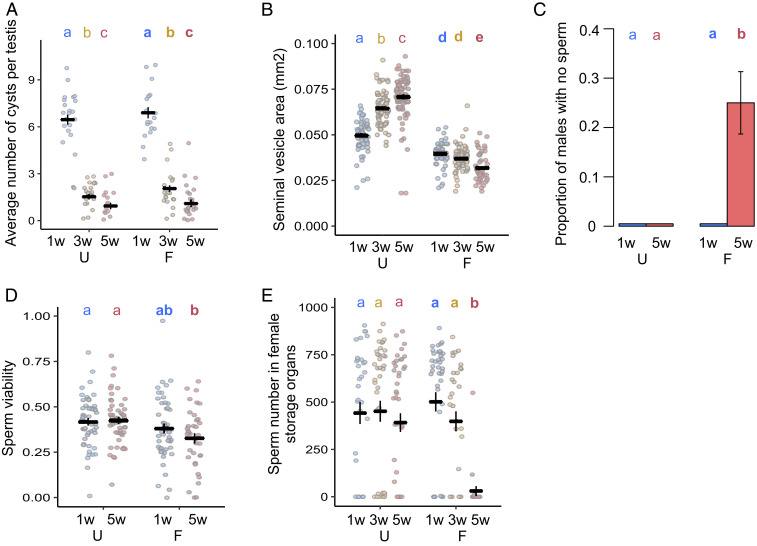
Aging and mating impact sperm production and transfer. (*A*) Average number of mature germline cysts per testis (a measure of sperm individualization rate) (age: χ^2^_2_ = 223.78; *P* < 0.0001; mating: χ^2^_1_ = 1.334; *P* = 0.119; age and mating interaction: χ^2^_2_ = 0.772; *P* = 0.496) (*n* = 16–22). (*B*) Seminal vesicle area (mm^2^) (age and mating interaction: *F*^2^_2_ = 68.494; *P* < 0.0001) (*n* = 49–79). (*C*) Proportion of males with no evidence of sperm within the seminal vesicle (*n* = 47–51). (*D*) Sperm viability 10 min after removal from the seminal vesicles (male age: χ^2^_1_ = 0.308; *P* = 0.871; mating group: χ^2^_1_ = 121.59; *P* = 0.001; interaction between male age and mating group: χ^2^_1_ = 13.537; *P* = 0.282) (*n* = 36–51). (*E*) Number of GFP fluorescent sperm heads in female sperm storage organs 90 min after mating starts (*binomial*: age and mating interaction: χ^2^_2_ = 13.417; *P* = 0.0012; *count*: age and mating interaction: χ^2^_2_ = 726.46; *P* = 0.0062) (*n* = 22–43). Results are shown as means ± SEM. Differences at *P* < 0.05 within mating groups and age categories are represented as different letters.

We next examined the testes and sperm of aging males to explore their role in male reproductive aging. Consistent with previous evidence of declining rates of spermatogenesis with age in flies (43), we found that the number of germline cysts in the final individualization stage of spermatogenesis (44) declines substantially as males age (Fig. 4*A*). Strikingly, this decline occurs at indistinguishable rates in U and F males. This suggests that males undergo a chronological decline in sperm production which is invariant to mating activity, a surprising finding given that sperm production rate is known to be malleable. For example, males elevate sperm production in response to the presence of rivals (45), and testis germline stem cell maintenance responds plastically to nutrition (46).

In the absence of mating, the size of the seminal vesicles (where mature sperm are stored) increases in U males but decreases in F males (Fig. 4*B*). This suggests that, like Sfps in the accessory glands, sperm stores accumulate in U males, despite the declining rate of sperm production. However, in contrast to Sfps, males are unable to sufficiently replenish sperm when they mate frequently throughout life, leading to depletion of sperm stores and high incidences of spermlessness in the seminal vesicles of Old-F males (Fig. 4 *B* and *C*). F males also have lower sperm viability, independent of age class, suggesting that regular copulation leads to reduced sperm quality, even if only for a few days (Fig. 4*D*). As might then be expected, we found a significant reduction in the number of sperm present in sperm storage organs (seminal receptacle and spermatheca) of females mated to Old-F males, relative to all other treatments, although there were also nonsignificant downward trends for both Old-U and 3-wk-F males (Fig. 4*E*).

The fact that Old-F males are sperm depleted but show no evidence of decline in Sfp quantity indicates that a mismatch develops in the relative capacity to produce sperm and Sfps (*SI Appendix*, Fig. S9). Mismatches, in general, might be expected to arise if there are fundamental differences in aging patterns between germline and soma as expected under theories, such as the disposable soma. In the short term, when males mate several times in rapid succession, seminal fluid rather than sperm is thought to limit fertility in male *Drosophila* and other insects (47), but our data show that, over the long term, Sfp replenishment capacity remains strong and is little affected by age. Sperm production declines, therefore, seem to be a major contributor to age-related infertility in sexually active *Drosophila* due to the loss of replenishment capacity. This pattern is, however, not necessarily predicted under the disposable soma hypothesis whereby resources should be prioritized for the germline over the soma. Superficially, therefore, we might expect seminal fluid to show declines before sperm. However, in the wild, or under normal laboratory conditions, male reproductive aging patterns would likely fall in between the extremes used in our experiments (U and F) meaning that such a striking mismatch between sperm and seminal fluid aging would be less common. Our data suggest that males make a finite lifetime investment in sperm that begins with high production but declines with age. This might adaptively free up resources to invest in other traits, such as mate attraction and survival, as males age. For example, given that sperm take 10 d to generate from start to finish (48) it may, on average, pay males to reduce investment in sperm production as they age because of diminishing life expectancy, rather than continuing to make costly sperm that will likely not complete development prior to death. Alternatively, the costs of initial sperm production may be borne through reduced production capacity later in life. Nonetheless, our data indicate that the relative importance of seminal fluid versus sperm factors in declining ejaculate performance with age will likely depend on the mating success of individual males with highly successful males potentially running out of sperm; a pattern seen previously in wild Soay Sheep (5). Sperm production declines are also prominent in humans: the daily rate approximately halves between the ages of 20 and 60 (49). A prime candidate for interventions to delay age-related ejaculate deterioration is, therefore, in the mechanisms that lead to declining sperm production.

### Changes in Reproductive Aging with Lifespan Extension.

**Fig. 5.**
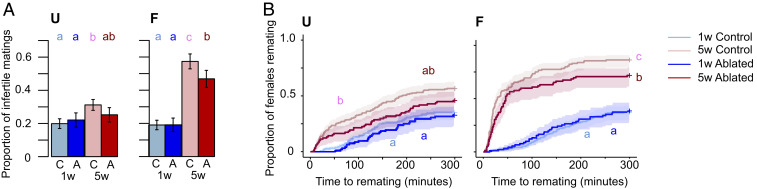
Manipulation of the insulin signaling pathway ameliorates ejaculate deterioration in Old-F males. (*A*) The proportion of infertile matings (*U:* age: χ^2^_1_ = 10.413; *P* = 0.001; line: χ^2^_1_ = 0.437; *P* = 0.508; age and line interaction: χ^2^_1_ = 1.941; *P* = 0.164; *n* = 95–202) (*F:* age: χ^2^_1_ = 115.83; *P* < 0.0001; line: χ^2^_1_ = 5.433; *P* = 0.0198; age and line interaction: χ^2^_1_ = 3.684; *P* = 0.055; *n* = 94–189). (*B*) Female remating latency (*U:* age: χ^2^_1_ = 28.927; *P* < 0.0001; line: χ^2^_1_ = 3.602; *P* = 0.058; age and line interaction: χ^2^_4_ = 0.660; *P* = 0.416; *n* = 92–198) (*F:* age and line interaction: χ^2^_1_ = 4.727; *P* = 0.03; *n* = 91–186). “C” stands for control and “A” stands for ablated lines. Results are shown as means ± SEM. Shaded areas are confidence intervals at the 0.15 level. Differences at *P* < 0.05 within lines and age categories are represented as different letters.

Manipulations of the insulin signaling pathway can extend lifespan in a broad range of taxa (50, 51), but it is unclear if lifespan extension results in a trade-off with male reproductive function—the germline, or ejaculate as a whole—or whether it could provide cobenefits to late-life ejaculate health. Given that we found very different impacts of age on sperm and seminal fluid, especially under high mating regimes, any improvements in late-life ejaculate function would potentially need to benefit all components of the ejaculate. To test these ideas, we used males in which the *Drosophila* insulin-like peptide (dilp)-producing median neurosecretory cells were ablated late in development (52) (hereon “ablated males”) and confirmed that these males display increased survival (*SI Appendix*, Fig. S10). We found clear evidence that the ablated males have improved ejaculate performance. Old-F ablated males are significantly less likely to be infertile and significantly better at suppressing female remating than Old-F control males (Fig. 5). Similar trends were seen in the U males, suggesting that, while the delay to reproductive senescence is stronger under frequent mating, the benefits may be more general. We did not detect any significant differences between ablated and control males in offspring production and paternity share (*SI Appendix*, Fig. S11). We also found that old ablated males are more likely to successfully copulate than controls, although the effect was more striking in the F treatment (*SI Appendix*, Fig. S12). These results show that inhibition of the insulin signaling pathway, in addition to extending lifespan, can ameliorate at least some aspects of age-related loss of mating and ejaculate performance. This result is in line with previous work showing that offspring production and lifespan are maximized at the same intake of nutrients (a high intake of carbohydrates but a low intake of proteins) in males, contrary to the prediction of an obligate trade-off between lifespan and reproduction (53).

This result apparently contrasts with the effects of rapamycin in mice, which extends lifespan, but causes testicular degeneration (20), the only other study to date where an aspect of ejaculate health has been assessed under experimental lifespan extension in males. There are numerous possible explanations for the discrepancy between the mouse study and our findings in flies, including differential action of insulin versus rapamycin pathways in male reproductive organs, or of drug treatment versus gene knockdown, as well as taxon-specific responses. Whether the improvements to late-life fertility in ablated males result from direct influences of reduced insulin activity in male reproductive tissues or as part of overall organismal health (or both) remain to be elucidated.

## Conclusion

Our *Drosophila* study provides a uniquely comprehensive exposition of the effect of male age on sperm and the seminal proteome and links these changes to a suite of fitness-related ejaculate performance phenotypes. Our data show that both the quantity and the quality of sperm and seminal fluid proteins can contribute to the age-related decline in male ejaculate performance, but that the role of these different factors is dependent on mating history. Thus, while sperm is a key factor in age-related infertility in a high-mating environment—in contrast to germline prioritization over soma that might be expected under the disposable soma hypothesis—the seminal fluid shows more generalized qualitative changes. The importance of seminal fluid to multiple facets of reproductive health is becoming increasingly apparent (9). For instance, human seminal plasma posttranslational modifications are now seen as potential biomarkers for assessment of male fertility (54). Thus, understanding seminal fluid health in aging males, across a range of taxa, represents a key future challenge. In agreement with the human seminal plasma work (54), our data highlight seminal protein quality as a potentially significant but currently understudied factor in ejaculate health. Finally, our results indicate that organism-level insulin signaling is not just a mediator of the male lifespan, but also of the male reproductive healthspan. Given the high degree of conservation in nutrient-sensing pathways and the considerable overlap in the process of spermatogenesis, Sfp-producing cells, the categories, and function of Sfps across taxa (55, 56), our data raise the possibility that interventions to promote healthy aging could be potentially co-opted to ameliorate male age-related infertility.

## Materials and Methods

### Stock and Fly Maintenance.

All flies were maintained at 25 °C on a 12:12 light–dark cycle and fed Lewis medium supplemented with ad libitum live yeast. Flies were reared using a standard larval density method by placing ∼200 eggs on 50 mL of food in 250-mL bottles. Virgins were collected on ice anesthesia within 8 h of eclosion and were randomly assigned to their experimental group. We used the *Dahomey* (*Dah*) wild-type stock, and all mutant lines were backcrossed into this background. Spermless males were *son*-*of*-*tudor* (30). Males of the same genetic background, where mothers did not have the *tudor* mutation, were used as controls. Fluorescently tagged sperm were from a *Ub-GFP* (green fluorescent protein) line (57). Ablated males were *UAS-rpr* > *InsP3GAL* (51, 58). Females were either *Dah* (in male aging vials) or *sparkling* (*spa*) (in phenotypic assays). *spa* provides a recessive phenotypic marker for paternity assignment. Males used in sperm competition assays were also *spa*.

#### Experimental design.

##### Wild-type males.

Upon eclosion, experimental virgin *Dah* males were allocated to one of two social group treatments. They were either housed in single sex groups of 12 (U), or three virgin *Dah* males were placed with nine virgin *Dah* females (F) (31). Males from three age classes were used: 1w, 3w, and 5w old. Only 1w and 5w age classes were used in the egg-laying assays. The U flies were transferred once per week, and the F flies were transferred twice a week to fresh vials using light CO_2_ anesthesia at each transfer. During the transfers, dead or escaped females were replaced with similarly aged females. To minimize female coaging effects in the Old F group, females were replaced at 3 wk with virgin 3- to 5-d-old females, reared using the same procedures as above. To minimize density effects on mating opportunity in the F vials, two vials of the same treatment were merged when a single male was left in a vial owing to previous mortality or censoring. The males from F were merged into single sex groups of 10–12 males 4–5 d before assaying in order to provide a consistent period of sexual rest prior to the assay point.

##### Spermless males.

In the spermless experiments, F vials had one experimental *son-of-tudor* male, two *spa* males, and nine virgin *Dah* females: the fertile *spa* males were provided to ensure that females were fertilized and refractory to provide consistency with other experiments. The controls in the spermless experiment had one control for the *tudor* male, two *spa* males, and nine virgin *Dah* females. Only 1w- and 5w-age classes were used in the spermless, insulin-mutant, and sperm viability experiments.

#### Mating experiments.

##### Wild-type males.

The day before the mating assays, 3- to 4-d-old virgin *spa* females were placed individually in vials. On the day of mating assays, experimental *Dah* males from each aging and mating treatment were added to the individually housed female vials and were given 5 h to mate. Matings and associated parameters (mating latency, mating duration, and mating success) were recorded. The mated females were allowed to lay eggs for 2 d, and the emerging offspring were counted to measure male fertility and offspring production. Then, the females were transferred into a new vial with two 6- to 7-d-old virgin *spa* males and were given the opportunity to remate once for up to 5 h. Matings and associated parameters (remating latency and remating success) were recorded. The remated females were allowed to lay eggs for an additional 2 d, and the emerging offspring were phenotyped and counted to measure paternity share.

The newly mated *Dah* males were flash frozen in liquid nitrogen 30 min after the start of the mating. More experimental *Dah* males were flash frozen in liquid nitrogen without being exposed to females. We repeated this experiment to produce four independent biological replicates. We thawed flash frozen males and dissected their accessory glands and ejaculatory ducts on ice in phosphate-buffered saline (PBS) buffer (27). Nineteen reproductive glands from males of the same treatment and replicate were pooled in 25-μL PBS buffer on ice and sent for label-free quantitative proteomics sample preparation.

##### Wild-type males for egg-laying assays.

The mating assay setup was the same for the male assays. Immediately after copulation, the mated females and a number of 6-d-old virgin females (as controls) were transferred to yeast pasted vials and allowed to lay eggs for 1 d. The following day we counted the number of eggs laid in each vial. One day later we counted the number of hatched eggs in each vial. The emerging offspring and dead pupae were counted to measure the viability of hatched eggs developing to pupae or adulthood. The experiment was repeated one more time to have two independent biological replicates.

##### Insulin-mutant males.

Ablated male mating phenotypes were examined as above except experimental males were *UAS-rpr* > *InsP3GAL*, *InsP3GAL*/+, and *UAS-rpr*/+. The experiment was repeated two more times to have three independent biological replicates. It was run over 2 d in each replicate, and some of the experimental 5w males and *spa* males that failed to mate in the first day were tested again on the second day, which we controlled for in the statistical analyses.

##### Spermless males.

The mating assay setup was the same for the spermless male assays, except that experimental *son-of-tudor* and fertile control males were added to the female vials and were given 4 h to mate. Immediately after copulation, half of the mated females and a number of 6-d-old virgin females (as controls) were transferred to new vials with two 4-d-old virgin *spa* males and given the opportunity to mate for 5 h. The other half of the mated females and a number of 6-d-old virgin females (as controls) were transferred to yeast pasted vials and allowed to lay eggs for 1 d. The following day we counted the number of eggs laid in each vial.

#### Sperm number and accessory gland measurements.

##### GFP sperm males.

The mating assays were conducted in the same way as for the wild-type *Dah* male experiments except that *Ub-GFP* males were used. The mated males were flash frozen in liquid nitrogen 30 min after the start of the mating. Additional experimental *Ub-GFP* males were flash frozen in liquid nitrogen without being exposed to females. We thawed flash frozen males and dissected their accessory glands and seminal vesicles on ice in PBS buffer. We imaged and measured the size of each seminal vesicle and accessory gland using a microscope calibration slide and ImageJ. The mated females were flash frozen in liquid nitrogen 90 min after the start of the mating (57). We thawed flash frozen females and dissected their spermatheca and seminal receptacles on ice in PBS buffer. We imaged and counted the number of sperm in both tissues using a fluorescence microscope and ImageJ.

##### Wild-type males.

To examine spermatogenesis, measured as a number of germline cysts, adult testes of *Dah* males were dissected in PBS and fixed in 5% formaldehyde for 20 min at room temperature (RT), washed in PBS + 0.1% Triton X-100 for 15–20 min, and stained with rhodamine-phalloidin (0.1 μM, Sigma-Aldrich, P1951) and DAPI (10 μM, Invitrogen D1306) for 20 min at RT. Images were captured using a Zeiss LSM510 Confocal or an Olympus IX-81 motorized inverted microscope with a XM-10 monochrome camera (44).

To test sperm viability, *Dah* males were dissected in ice-cold PBS buffer. The observer was blinded to the treatment to avoid bias (59). The seminal vesicles were dissected from the testes and transferred to a 2.5-μL drop of PBS buffer on a new microscope slide and punctured to release sperm. The sample was covered to prevent evaporation and left for 5 min. Then, 1.25 μL of LIVE/DEAD stain (Thermo) was added, and sperm viability was scored (42). For each slide, four regions were imaged using a fluorescence microscope under both green and red filters, giving four pairs of images per slide. The images were processed in ImageJ, and sperm viability for each sample was calculated as the proportion of sperm that were live. The experiment was repeated one more time to have two independent biological replicates. The imaging was performed over 4 and 5 d in each replicate, respectively.

Then, we tested the effect of seminal fluid produced by Young- and Old-U and -F males on sperm viability. The accessory glands of experimental males were dissected and transferred to a 2.5-μL drop of PBS buffer on a new microscope slide and punctured to release seminal fluid. Another 1w-virgin male (standard) was dissected, and the seminal vesicles were transferred to the same slide as the accessory glands. The seminal vesicles were punctured to release sperm; the seminal fluid and the sperm were mixed briefly with a pin. A proportion of the sperm samples (43 out of the total 210 samples) were mixed briefly with a pin without adding seminal fluid as controls. The sample was covered to prevent evaporation and left for an hour to give time for viability differences between treatments to develop (42). Then, 1.25 μL of LIVE/DEAD stain was added, and sperm viability was scored. The imaging was performed as detailed above.

#### Label-free quantitative proteomics.

##### Wild-type males.

All samples described above were stored at −80 °C until sample preparation for proteomic analysis. The samples were macerated with a clean pestle and washed with 25 μL of Pierce RIPA buffer. Then, they were digested using the standard gel-aided sample preparation protocol as described previously (31, 60). Peptide samples were analyzed on a liquid chromatography tandem mass spectrometry platform, a Q-Exactive HF mass spectrometer (Thermo), and processed as described previously (31).

#### Western blot assays.

##### Wild-type males.

Single males were ground in 10 μL of sample buffer with a plastic pestle in 0.6-mL Eppendorf tubes. The samples were boiled for 5 min and spun for 2 min at 15,000 rpm at RT. They were loaded into 13 × 13-cm 5–15% gradient polyacrylamide (Amresco catalog no. M157) sodium dodecyl sulfate (SDS) gels with a 4% polyacrylamide stacking SDS gel. Gels were run at RT for 30 min at 110 V, then moved to 4 °C and run for 5 h at 150 V until the dye front was ∼9 cm from the stacking gel. The gels were wet transferred to a polyvinylidene difluoride (Millipore catalog no. IPFL00010) membrane overnight at 4 °C and 40 V. Membranes were dried for, at least, 30 min to cross-link protein, then rewet with 100% MeOH, and blocked with 5% milk in 1× tris-buffered saline, 0.1% Tween 20 (TBST) for 1 h at RT. Primary antibodies (polyclonal anti-rabbit custom made in the laboratory) were diluted in 1% milk in 1× TBST (0.1% Tween 20) for, at least, 2 h at RT, or overnight at 4 °C. Membranes were rinsed 2× then washed 4× for 10 min each with 1× TBST (0.1% Tween 20). The secondary antibody was goat anti-rabbit immunoglobulin G (H+L) horseradish peroxidase (Jackson Immuno Research catalog no. 111–035-003). Membranes were incubated for 1 h in a secondary antibody diluted 1:2,000 in 5% milk in 1× TBST (0.1% Tween 20) for 1 h at RT. Rinses and washes were repeated as above. Membranes were detected with Pierce ECL2 (Fisher catalog no. PI80196) and developed for 5 min at RT, then chemiluminescence was measured on a Typhoon scanner. Membranes were stripped with β-mercaptoethanol stripping buffer for 50 min at 50 °C with shaking, then rinsed three times for 5 min each with 1× TBS, before blocking (as above) and adding an additional primary antibody. Primary antibody dilutions were 1:500 for 11864 Semp1, 1:5,000 for Acp26Aa, 1:30,000 for Acp36DE, 1:2,000 for Acp62F, 1:1,000 for CG9997, and 1:5,000 for Sex peptide.

### Data Analysis.

Data were analyzed using RStudio 1.1.383 (61).

#### Wild-type males.

The proportion of survivors was compared between treatments using generalized linear models (GLMs) with a binomial error distribution corrected for overdispersion. The mating latency and remating latency of females were analyzed using the *survival* package and a Weibull distribution. Mating duration was analyzed using a Gaussian distribution with an identity link function. The proportion of infertile males (those producing zero offspring) was tested using GLMs with a binomial error distribution. The number of offspring was analyzed for fertile matings (i.e., those that produced, at least, one offspring), and was tested using GLMs with Poisson error distribution corrected for overdispersion. Paternity share was analyzed using GLMs with a binomial error distribution corrected for overdispersion. The initial model included male age, mating history, their interaction, and replicate number as fixed effects. In all analyses, model selection was performed by backward stepwise elimination; nonsignificant (*P* > 0.05) variables were eliminated from the model to arrive at the minimal adequate model. However, replicate number was kept in the minimal model to control for this variation.

For the label-free quantitative proteomic dataset, only proteins identified with at least two unique peptides were included in the final dataset. Further details on the analyses can be found in ref. 31. We focused our analyses on previously identified Sfps (31, 34). Age and mating-related Sfp abundance differences and transferred Sfp abundance differences were analyzed for each Sfp separately using linear mixed effect models. Here, the initial model included male age, mating history, their interaction as fixed effects, and replicate number as a random effect. Model selection was performed by backward stepwise elimination, and the resulting *P* values were corrected for multiple testing using the Benjamini–Hochberg procedure. Age and mating-related Sfp abundance differences and transferred Sfp abundance differences were also analyzed for all of the Sfps together using linear mixed effect models. Here, the initial model included male age, mating history, their interaction as fixed effects, protein name, and replicate number as random effects. We inferred the abundance of Sfps transferred to the female by subtracting the Sfp abundance of newly mated males from males not exposed to females within the same treatment and replicate (31). The heatmaps were made using a Pearson correlation distance metric and plotted using the *pheatmap* package, and the data were mean centered (standardized) for each protein for better visualization. The lineplots were made using the *ggplot2* package. Age and mating-related compositional changes in the seminal fluid proteome and the transferred seminal fluid proteome were assessed using principal component analyses and linear mixed effect models. Again, the initial model included male age, mating history, their interaction as fixed effects, and replicate number as a random effect.

Sperm viability inside the seminal vesicles was analyzed using two GLMs. The first one modeled the presence/absence of any sperm using a binomial error distribution. The second one modeled the percentage data (percentage of live sperm within a sample) using a Poisson error distribution corrected for overdispersion. Sperm viability following mixing with seminal fluid from a different male was analyzed using GLMs with a binomial error distribution corrected for overdispersion. The initial model included donor type (seminal fluid from Young-U, Old-U, Young-F, Old-F males, or no seminal fluid), replicate number, and day of experiment as fixed effects. Replicate number and day of experiment were kept in the minimal model as we wanted to control for the variation introduced by these factors. The average number of cysts per testis was analyzed using GLMs with Poisson error distribution corrected for overdispersion.

#### Wild-type males for egg-laying assays.

The number of eggs laid by virgin females or females mated to wild-type males were analyzed using two GLMs. The first one modeled the presence/absence of nonzero values using a binomial error distribution. The second one modeled the nonzero count data using a Poisson error distribution corrected for overdispersion. In each analysis, the initial model included female mating treatment (Young-U mating, Old-U mating, Young-F mating, Old-F mating, and virgin) and replicate number as a fixed effect. Egg hatchability, hatched egg-to-pupae, and hatched egg-to-adult viability were tested using GLMs with a binomial error distribution corrected for overdispersion. Here, the initial model included male age, mating history, their interaction, and replicate number as fixed effects.

#### Spermless males.

The number of eggs laid by virgin females or females mated to spermless or fertile control males were analyzed using a Poisson error distribution corrected for overdispersion. The mating latency of females was analyzed using a Weibull distribution. The data were analyzed separately for U and F groups. The initial model included female mating treatment (young spermless mating, old spermless mating, young control mating, old control mating, and virgin) as a fixed effect.

#### GFP sperm males.

The number of sperm in female sperm storage organs was analyzed using two GLMs. The first one modeled the presence/absence of nonzero values using a binomial error distribution. The second one modeled the nonzero count data using a Poisson error distribution corrected for overdispersion. In each analysis, the initial model included male age, mating history, their interaction, and day as fixed effects. Day was kept in the minimal model to control for the factor. The size of accessory glands and seminal vesicles was analyzed using a Gaussian distribution with an identity link function. Here, the models included male age, mating history, and their interaction as fixed effects.

#### Insulin-mutant males.

For the ablated male experiments, the proportion of survivors at the time of assay was compared between treatments using GLMs with a binomial error distribution corrected for overdispersion. The mating latency and remating latency of females were analyzed using a Weibull distribution. The proportion of infertile matings was analyzed using GLMs with a binomial error distribution. The number of offspring was analyzed for fertile matings using GLMs with Poisson error distribution corrected for overdispersion. Paternity share from first mating was analyzed using GLMs with a binomial error distribution corrected for overdispersion. The data were analyzed separately for U and F groups. The initial model included male age, male line, their interaction, replicate number, day, and whether the vial had recycled males. However, replicate number, day, and whether the male was recycled were kept in the minimal model to control for these factors. Out of the 24 models we ran, in only four, the two controls had significantly different responses (*SI Appendix*, Fig. S3). We, therefore, merged the two control genotypes as a single control to simplify subsequent analyses.

## Supplementary Material

Supplementary File
