# Supplementary material for: Male reproductive aging arises via multifaceted mating-dependent sperm and seminal proteome declines, but is postponable in Drosophila
Source: Proc Natl Acad Sci U S A. 2020 Jul 1;117(29):17094–103. doi: 10.1073/pnas.2009053117 (PMC7382285; doi:10.1073/pnas.2009053117)
Supplement: Supplementary File [file pnas.2009053117.sapp.pdf]

Supplementary Information for

Male reproductive ageing arises via multifaceted mating-dependent sperm and seminal proteome declines, but is postponable in *Drosophila*

Irem Sepil, Ben R Hopkins, Rebecca Dean, Eleanor Bath, Solomon Friedman, Ben Swanson, Harrison Ostridge, Lucy Harper, Norene A Buehner, Mariana F Wolfner, Rebecca Konietzny, Marie-Laëtitia Thézénas, Elizabeth Sandham, Philip D Charles, Roman Fischer, Josefa Steinhauer, Benedikt M Kessler, Stuart Wigby

Correspondence to: irem.sepil@zoo.ox.ac.uk

This PDF file includes:

Figs. S1 to S12
Tables S1 to S3

www.pnas.org/cgi/doi/10.1073/pnas.2009053117

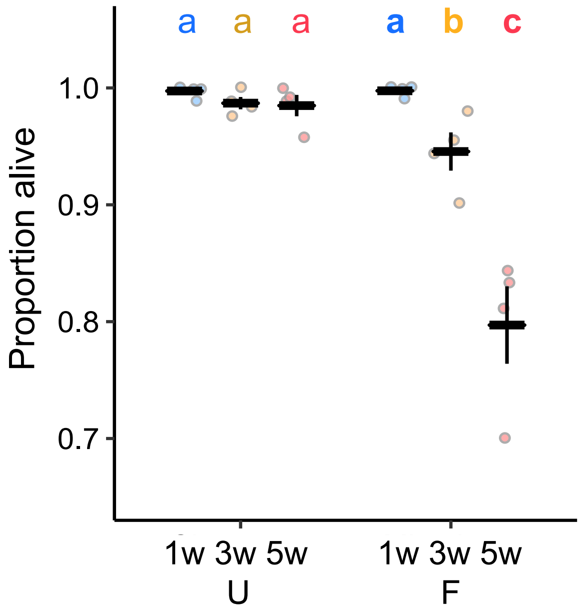

Fig. S1: Old-F males are less likely to survive the experiment. Age: χ^2_2= 120.36; p< 0.0001; mating: χ^2_1= 92.732; p< 0.0001; age and mating interaction: χ^2_2= 6.246; p= 0.170 (n= 384 - 444, pooled from four replicates). Results are shown as means ± SEM. "U" stands for unmated and "F" stands for frequently mated males. Differences at p < 0.05 within mating groups and age categories are represented as different letters.

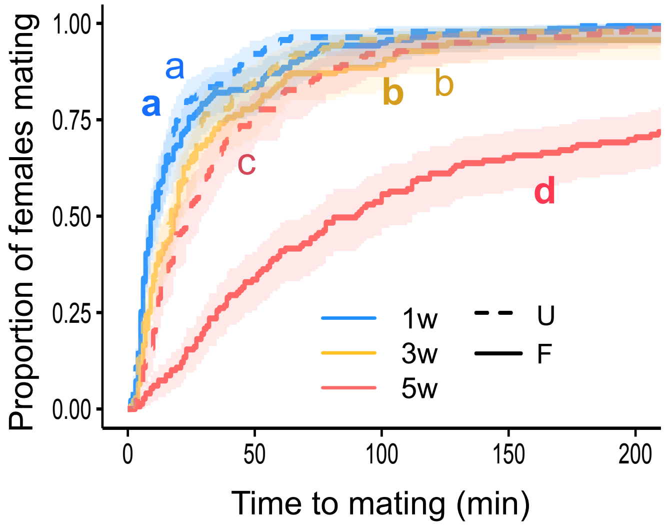

Fig. S2: Old males show a significant reduction in copulation probability. Age and mating interaction: χ^2_2= 55.278; p < 0.0001 (n= 139-149). "U" stands for unmated and "F" stands for frequently mated males. Shaded areas are confidence intervals at 0.15 level. Differences at p < 0.05 within mating groups and age categories are represented as different letters.

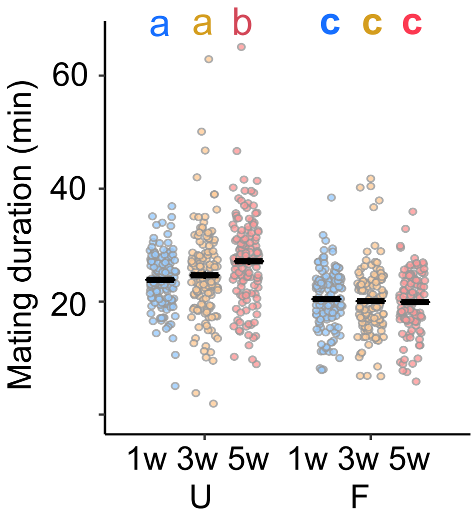

Fig. S3: Old-U males show a significant increase in mating duration. Age and mating interaction: χ^2_2= 6.6462; p = 0.001 (n= 113-138). "U" stands for unmated and "F" stands for frequently mated males. Differences at p < 0.05 within mating groups and age categories are represented as different letters.

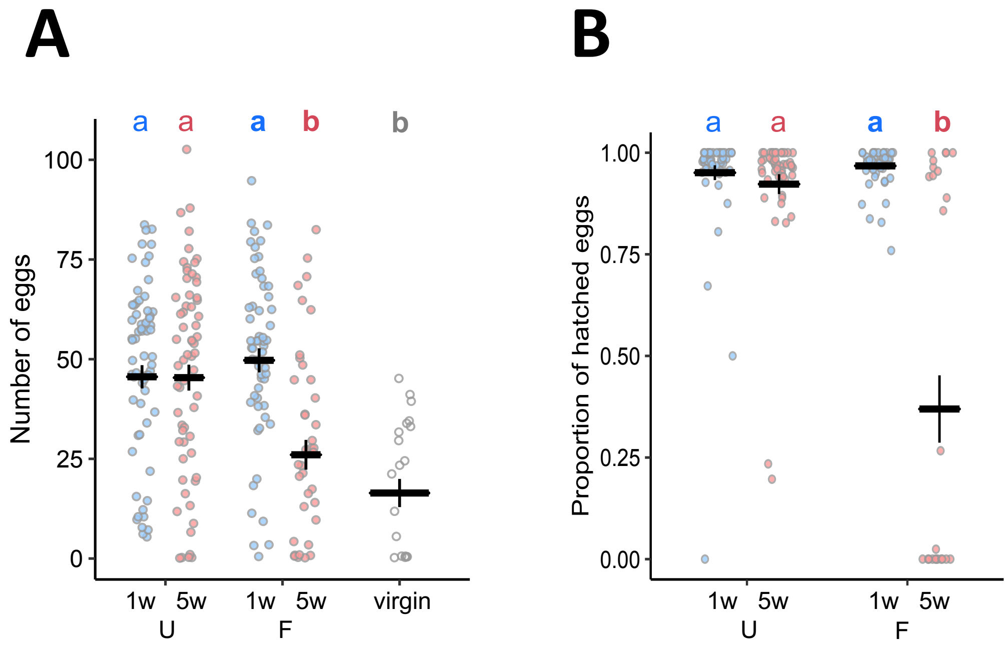

Fig. S4: (A) Females mated to Old-F males lay fewer eggs - similar numbers as virgin females (*binomial*: female mating treatment: χ^2_4= 31.082; p< 0.0001; *count*: female mating treatment: χ^2_4= 334.46; p< 0.0001) (n= 23-67). **(B)** The eggs from Old-F matings are less likely to hatch (age and mating interaction: χ^2_1= 202.26; p< 0.0001) (n=32-63). "U" stands for unmated and "F" stands for frequently mated males. Differences at p < 0.05 within treatments are represented as different letters.

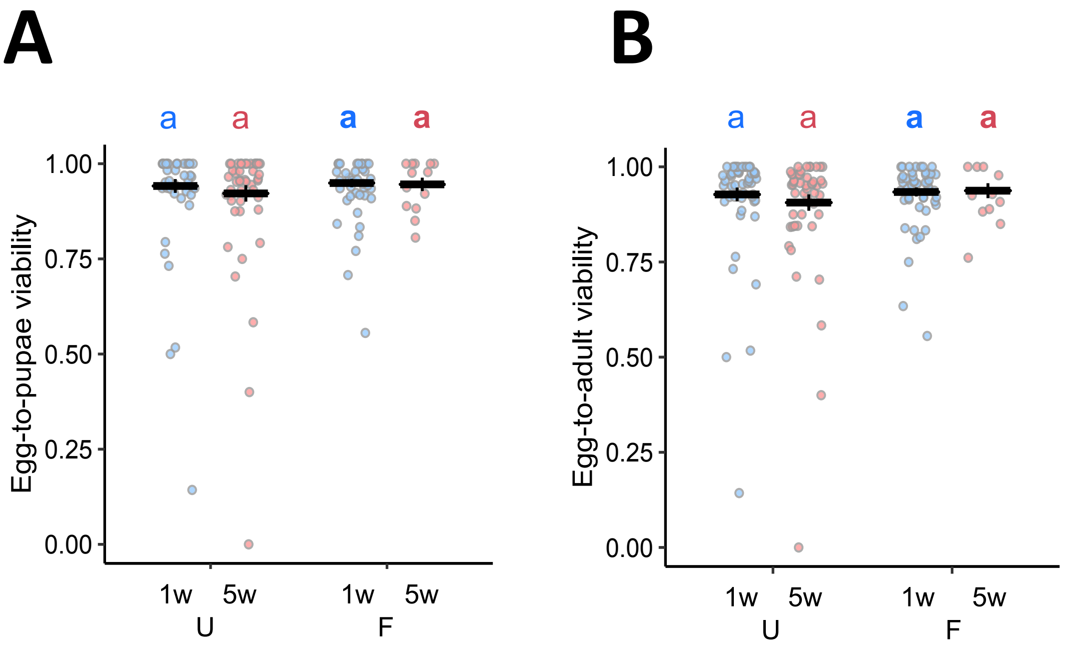

Fig. S5: Male age and mating history do not have a significant effect on **(A)** egg-to-pupae viability of hatched eggs (male age: χ^2_1= 8.822; p= 0.284; mating group: χ^2_1= 1.168; p= 0.698; interaction between male age and mating group: χ^2_1= 0.280; p= 0.85) (n= 14-62); **(B)** egg-to-adult viability of hatched eggs (male age: χ^2_1= 7.353; p= 0.277; mating group: χ^2_1= 0.759; p= 0.728; interaction between male age and mating group: χ^2_1= 0.976; p= 0.694) (n= 14-62). "U" stands for unmated and "F" stands for frequently mated males. Differences at $p < 0.05$ within mating groups and age categories are represented as different letters.

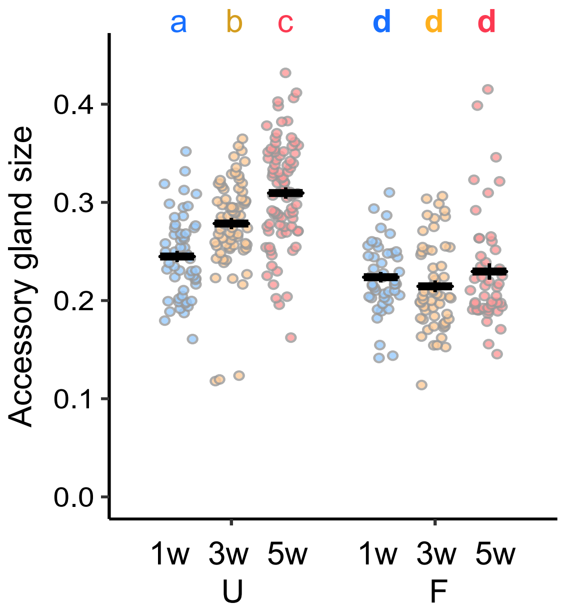

Fig. S6: Aging and frequent mating impacts on accessory gland area (mm^2). Age and mating interaction: $F^2_2 = 10.685$; $p < 0.0001$ (n= 49 - 84). “U” stands for unmated and “F” stands for frequently mated males. Results are shown as means ± SEM. Differences at $p < 0.05$ within mating groups and age categories are represented as different letters.

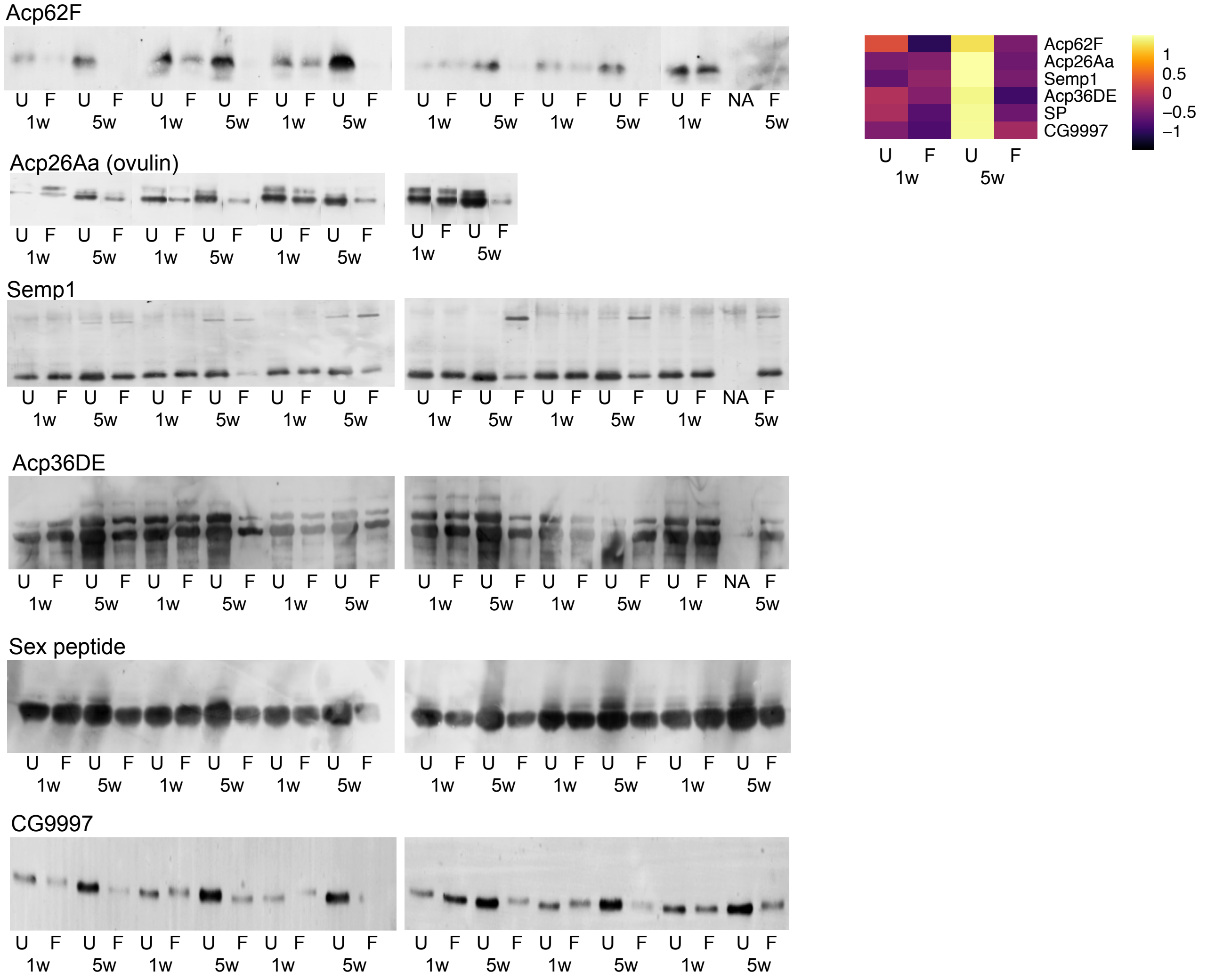

Fig. S7: The gel mobility of a number of functionally important Sfps (Acp62F, Acp26Aa, Semp1, Acp36DE, Sex peptide, and CG9997) as determined by Western blots, in 1w and 5w males from U and F groups. The abundance of each protein is predicted from the proteomic data and illustrated as a heatmap. Each lane is an individual male. “U” stands for unmated and “F” stands for frequently mated males.

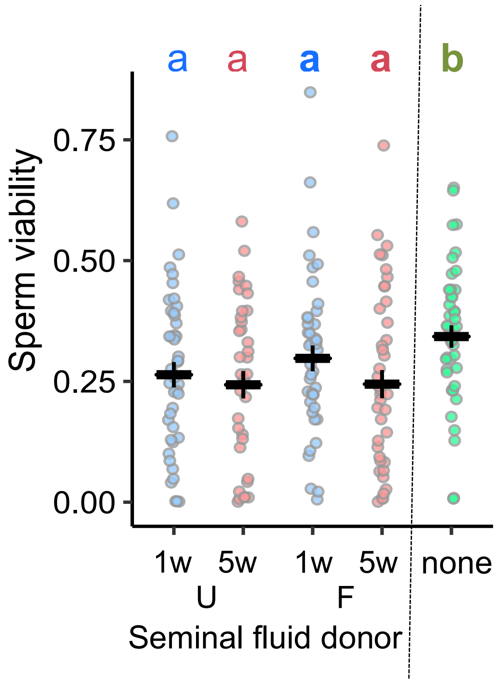

Fig. S8: No evidence that sperm viability 1 hour after removal from the seminal vesicles responds to the age and mating status of the male contributing the seminal fluid. Also no evidence that seminal fluid from a different male protects sperm. Donor type: $\chi^2_4= 264.96$; $p= 0.013$ ($n= 38 – 45$). “U” stands for unmated and “F” stands for frequently mated males. Results are shown as means ± SEM. Differences at $p < 0.05$ within between treatments are represented as different letters.

Male status	Male sperm and seminal fluid stores	Transferred ejaculate	Fertility	Seminal proteome composition	Seminal stimulation of females
Young	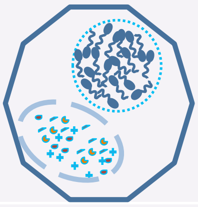	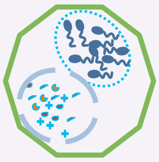	High (standard + competitive)	Baseline	High (fecundity + refractoriness)
Old (Unmated)	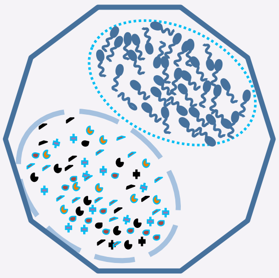	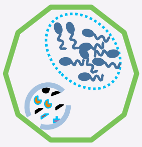	High (standard) Low (competitive)	Altered	Low (fecundity) High (refractoriness)
Old (Frequently mated)	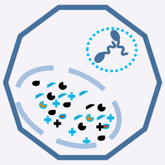	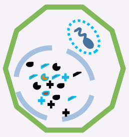	Low (standard + competitive)	Baseline	Low (fecundity + refractoriness)

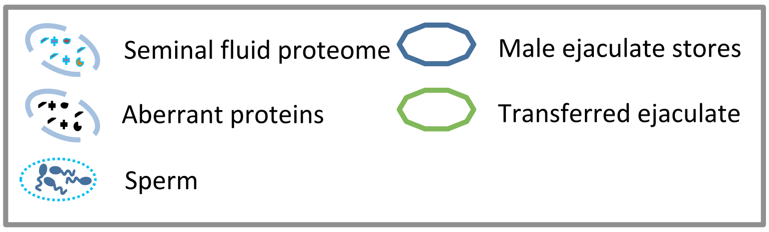

Fig. S9: Schematic summary of the impacts of ageing on the ejaculate.

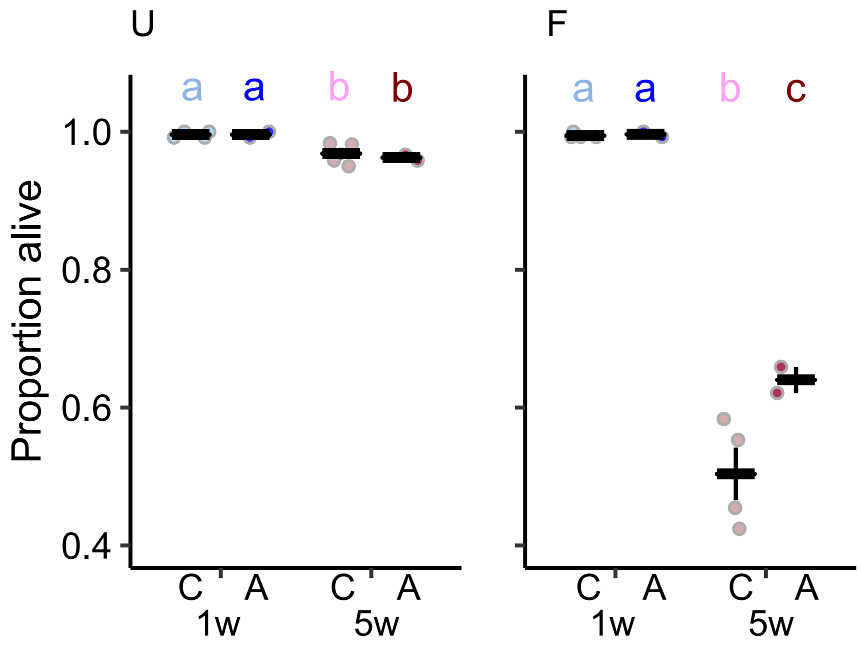

Fig. S10: Old-F ablated males (*UAS-rpr > InsP3GAL*) are more likely to survive the experiment compared to Old-F control males (*InsP3GAL*/+ and *UAS-rpr*/+). *U:* age: χ^2_1= 19.288; p< 0.0001; line: χ^2_1= 0.124; p= 0.692; age and line interaction: χ^2_1= 0.015; p= 0.895. *F:* age: χ^2_1= 556.52; p< 0.0001; line: χ^2_1= 12.648; p= 0.005; age and line interaction: χ^2_1= 0.013; p= 0.932 (n= 228 - 528, pooled from two replicates). "C" stands for control and "A" stands for ablated lines. "U" stands for unmated and "F" stands for frequently mated males. Results are shown as means ± SEM. Differences at $p < 0.05$ within lines and age categories are represented as different letters.

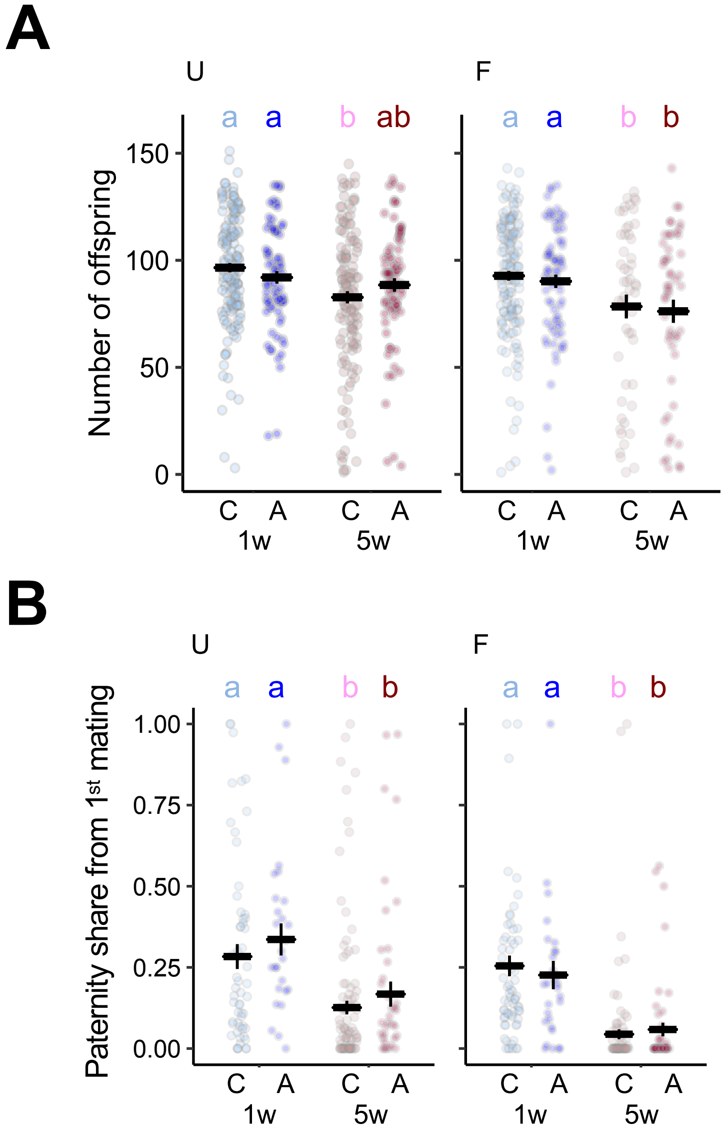

Fig. S11: No evidence of differential offspring production and paternity share between ablated (*UAS-rpr* > *InsP3GAL*) and control (*InsP3GAL*/+ and *UAS-rpr*/+) males as a response to age and mating status. **(A)** Offspring production (*U:* age: χ^2_1=133.4; p< 0.0001; line: χ^2_1=0.028; p= 0.953; age and line interaction: χ^2_1= 26.518; p= 0.0681) (*F:* age: χ^2_1=297.53; p< 0.0001; line: χ^2_1=0.102; p= 0.923; age and line interaction: χ^2_1=3.042; p= 0.6) (n= 51 – 153 pooled from three replicates). **(B)** Paternity share (*U:* age: χ^2_1= 681.12; p< 0.0001; line: χ^2_1= 2.582; p= 0.77; age and line interaction: χ^2_1= 39.15; p= 0.256) (*F:* age: χ^2_1= 1792; p< 0.0001; line: χ^2_1= 0.008; p= 0.986; age and line interaction: χ^2_1= 22.254; p= 0.365) (n= 30 – 112 pooled from three replicates). “C” stands for control and “A” stands for ablated lines. “U” stands for unmated and “F” stands for frequently mated males. Results are shown as means ± SEM. Differences at p < 0.05 within fly lines and age categories are represented as different letters.

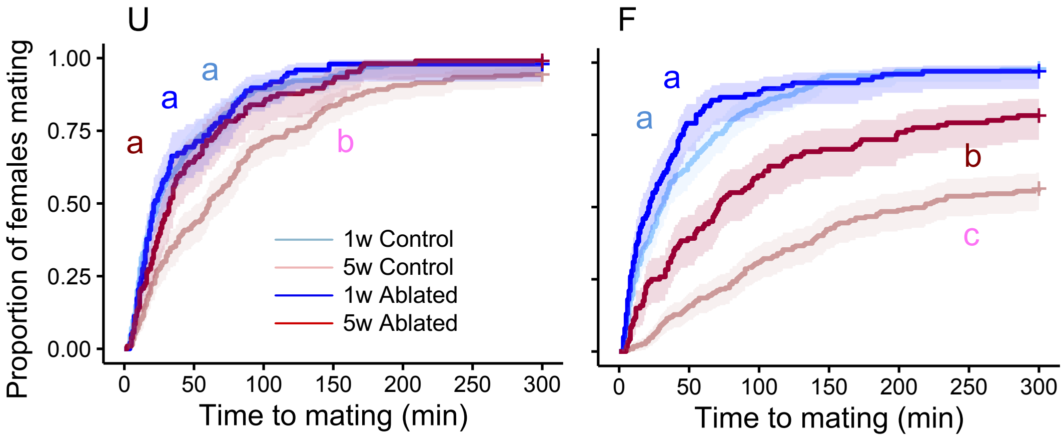

Fig. S12: Females are more likely to mate with Old-F ablated males (*UAS-rpr* > *InsP3GAL*) compared to Old-F control males (*InsP3GAL*/+ and *UAS-rpr*/+). *U:* age and line interaction: χ^2_1= 7.447; p= 0.006 (n= 98-213). *F:* age and line interaction: χ^2_1= 5.472; p= 0.019 (n= 100-227). "U" stands for unmated and "F" stands for frequently mated males. Shaded areas are confidence intervals at 0.15 level. Differences at $p < 0.05$ within mating groups and age categories are represented as different letters.

Table S1: The list of Sfps detected in this study and their functional categories. The abundance of 40 Sfps show a significant differential response to age and mating after false discovery rate (FDR) correction (*Age x Mating qval*) and have been listed in the order of significance. Each Sfps individual response to age and mating after FDR correction are also included (*Age qval* and *Mating qval* respectively).

Protein	*Age qval*	*Mating qval*	*Age x Mating qval*	*Functional Category*
CG3097	0.001939	0.000001	0.0007	Protease
CG10587	0.000328	0.000005	0.0010	Protease
regucalcin	0.002151	0.000001	0.0010	Calcium ion binding
CG31413	0.006683	0.000010	0.0014	Cell redox homeostasis
Sfp65A	0.000127	0.000006	0.0014	Unknown function
mfas	0.000000	0.000002	0.0015	Cell adhesion
Spn42Dd	0.000267	0.000001	0.0015	Protease inhibitor
CG31659	0.000025	0.000001	0.0028	Lipid metabolism
CG34002	0.000215	0.000024	0.0029	Unknown function
CG17093	0.005077	0.000004	0.0031	Lipid metabolism
CG17097	0.007977	0.000009	0.0031	Lipid metabolism
CG34051	0.000731	0.003108	0.0031	Unknown function
Semp1	0.003534	0.001941	0.0031	Protease
CG31883	0.215710	0.000001	0.0040	Unknown function
Sems	0.002151	0.000020	0.0040	Post-mating behaviour
SP	0.000328	0.000001	0.0040	Post-mating behaviour
lectin-46Ca	0.002152	0.000123	0.0045	Post-mating behaviour
Sfp24F	0.001384	0.000143	0.0048	Carbohydrate interactions
CG34130-RA	0.000122	0.000001	0.0060	Protease
Acp26Ab	0.001849	0.000076	0.0098	Post-mating behaviour
CG31418	0.004048	0.000004	0.0098	Unknown function
CG34129	0.008755	0.000010	0.0098	Protease
CG3640	0.000267	0.000008	0.0098	Unknown function
CG11608	0.032160	0.001008	0.0114	Lipid metabolism
Acp26Aa	0.012934	0.000495	0.0122	Post-mating behaviour
CG10284	0.000132	0.000008	0.0122	Defense/immunity
Acp53Ea	0.000328	0.000048	0.0127	Post-mating behaviour
lectin-29Ca	0.001661	0.000003	0.0129	Carbohydrate interactions
Spn28F	0.007086	0.000012	0.0129	Protease inhibitor
CG31419	0.001849	0.000004	0.0135	Unknown function
CG31680	0.000063	0.000003	0.0176	Unknown function
aqrs	0.001384	0.000002	0.0188	Post-mating behaviour
Obp56i	0.000671	0.000000	0.0214	Odorant binding
NUCB1	0.000024	0.000819	0.0220	Defense/immunity
CG17472	0.000711	0.025065	0.0251	Unknown function
CG17843	0.000671	0.000006	0.0259	Cell redox homeostasis
Sfp38D	0.001849	0.000008	0.0279	Unknown function
CG17575	0.574146	0.042919	0.0328	Post-mating behaviour
CG17919	0.025211	0.011913	0.0328	Defense/immunity

CG9997	0.000267	0.000029	0.0344	Post-mating behaviour
CG4847	0.014889	0.000161	0.0539	Protease
CG9168	0.011594	0.023971	0.0539	Catalytic activity
CG9519	0.011649	0.176250	0.0539	Cell redox homeostasis
Est-6	0.024980	0.595285	0.0539	Post-mating behaviour
CG10651	0.010840	0.011092	0.0565	Unknown function
CG17242	0.003266	0.022477	0.0565	Protease
Hexo2	0.001661	0.000002	0.0565	Carbohydrate interactions
Spn38F	0.079187	0.011565	0.0565	Defense/immunity
Spn75F	0.012124	0.004355	0.0583	Protease inhibitor
CG10730	0.493557	0.083393	0.0587	Catalytic activity
Acp36DE	0.270410	0.000243	0.0655	Post-mating behaviour
CG18284	0.000215	0.009889	0.0718	Lipid metabolism
lectin-30A	0.120793	0.000010	0.0718	Carbohydrate interactions
Acp53C14a	0.029885	0.000028	0.0725	Unknown function
CG11598	0.294351	0.006846	0.0725	Lipid metabolism
CG15116	0.015728	0.369419	0.0725	Cell redox homeostasis
CG15117	0.014297	0.000005	0.0725	Carbohydrate interactions
CG30395	0.855762	0.001899	0.0725	Unknown function
CG31684	0.071129	0.001050	0.0725	Lipid metabolism
lectin-46Cb	0.002413	0.000737	0.0725	Post-mating behaviour
Obp56g	0.138601	0.017418	0.0747	Odorant binding
CG1701	0.085978	0.054908	0.0821	Unknown function
CG9029	0.047172	0.095215	0.0839	Defense/immunity
betaTub85D	0.645682	0.042919	0.0862	DNA interactions
antr	0.002805	0.000010	0.1017	Post-mating behaviour
BG642312	0.372316	0.001851	0.1075	Post-mating behaviour
Spn28B	0.008040	0.000039	0.1075	Protease inhibitor
Sfp78E	0.077672	0.006576	0.1163	Unknown function
CG14034	0.048468	0.000031	0.1209	Lipid metabolism
CG2852	0.590456	0.004743	0.1209	Catalytic activity
NLaz	0.008446	0.023971	0.1379	Lipid metabolism
Acp53C14c	0.398788	0.000306	0.1536	Unknown function
Sfp23F	0.086745	0.002687	0.1536	Protease inhibitor
Spn77Bb	0.398788	0.776094	0.1548	Protease inhibitor
Obp22a	0.000731	0.000123	0.1622	Odorant binding
Sfp26Ad	0.016149	0.000474	0.1629	Unknown function
Acp76A	0.333139	0.069725	0.1715	Protease inhibitor
CG6071	0.147006	0.685423	0.1715	Protease
Ggt-1	0.138347	0.000160	0.1715	Protease
CG6690	0.045501	0.000517	0.1849	Cell redox homeostasis
Spn28Db	0.148625	0.000006	0.1986	Protease inhibitor
CG18067	0.000267	0.445143	0.2091	Unknown function
Phm	0.185761	0.095019	0.2143	Catalytic activity
CG10041	0.924033	0.011473	0.2274	Protease
CG10407	0.424137	0.007841	0.2274	Unknown function
Acp29AB	0.382685	0.000335	0.2289	Post-mating behaviour

CG18135	0.415179	0.559060	0.2989	Lipid metabolism
CG5162	0.012421	0.040758	0.3634	Lipid metabolism
Sfp51E	0.006425	0.000393	0.3674	Unknown function
Sfp33A3	0.020628	0.004146	0.3882	Catalytic activity inhibition
Obp56f	0.097002	0.006955	0.4605	Odorant binding
S-Lap7	0.187481	0.182767	0.5183	Protease
CG15641	0.012124	0.000790	0.5300	Unknown function
CG34034	0.859900	0.391358	0.5323	Unknown function
Sfp24C1	0.014889	0.012834	0.5323	Protease inhibitor
Mst57Dc	0.031189	0.000158	0.5393	Post-mating behaviour
BG642163	0.128093	0.012834	0.5524	Unknown function
Obp51a	0.464016	0.445143	0.5642	Odorant binding
alphaTub84B	0.001972	0.153894	0.6126	DNA interactions
CG11112	0.070414	0.004466	0.6126	Unknown function
Dup99B	0.038606	0.332900	0.6126	Post-mating behaviour
CG32833	0.016324	0.001256	0.6511	Protease
Spn77Bc	0.103226	0.026241	0.6905	Protease inhibitor
Sfp70A4	0.294351	0.066354	0.6974	Unknown function
CG11037	0.030346	0.000847	0.7095	Protease
CG30486	0.294351	0.006955	0.7620	Unknown function
CG31515	0.168643	0.022477	0.7620	Protease inhibitor
Met75Ca	0.487535	0.327324	0.7753	Unknown function
Sfp35C	0.110603	0.003618	0.8377	Unknown function
Npc2b	0.011594	0.332306	0.8645	Hormone metabolism
Acp62F	0.364605	0.004466	0.8903	Post-mating behaviour
CG15635	0.590456	0.370730	0.9536	Unknown function
Acp53C14b	0.193640	0.019589	0.9736	Unknown function
CG31704	0.528657	0.018633	0.9736	Unknown function
CG34033	0.028763	0.000847	0.9736	Unknown function
Obp56e	0.814145	0.000635	0.9741	Odorant binding
CG43145	0.291021	0.008410	0.9763	Protease inhibitor

Table S2: The list of ejaculatory-duct specific Sfps detected in this study and their functional categories. The top eight ejaculatory-duct specific Sfps clustered separately from the rest of the Sfps. Each Sfps individual response to age, mating and their interaction after FDR correction are also included (*Age qval*, *Mating qval* and *Age x Mating qval* respectively).

Protein	*Cluster*	*Age qval*	*Mating qval*	*Age x Mating qval*	*Functional Category*
CG34034	Yes	0.860	0.391	0.532	Unknown function
Obp51a	Yes	0.464	0.445	0.564	Odorant binding
Met75Ca	Yes	0.488	0.327	0.775	Unknown function
Spn77Bc	Yes	0.103	0.026	0.691	Protease inhibitor
Est-6	Yes	0.025	0.595	0.054	Post-mating behaviour
Dup99B	Yes	0.039	0.333	0.613	Post-mating behaviour
CG5162	Yes	0.012	0.041	0.363	Lipid metabolism
CG17242	Yes	0.003	0.022	0.057	Protease
Obp56g	No	0.139	0.017	0.075	Odorant binding
CG18067	No	0.000	0.445	0.209	Unknown function
Spn77Bb	No	0.399	0.776	0.155	Protease inhibitor
CG31704	No	0.529	0.019	0.974	Unknown function
NLaz	No	0.008	0.024	0.138	Lipid metabolism

Table S3: Pairwise comparisons between the two control genotypes (*InsP3GAL*/+ and *UAS-rpr*/+) within each age and mating treatment. These are 1w and 5w old U and F treatments. The response variables are the proportion of infertile matings, female latency to remating, the proportion of flies to survive the experiment, offspring production, paternity share of the first male and female latency to mating. "U" stands for unmated and "F" stands for frequently mated males. Differences at $p < 0.05$ are given in red.

Effect	*Treatment*	*Estimate*	*Standard error*	*T value*	*P value*
Infertile matings	1wU	-0.874	0.761	-1.147	0.251
	5wU	-0.104	0.353	-0.293	0.769
	1wF	-1.066	0.891	-1.196	0.232
	5wF	0.855	0.445	1.921	0.055
Remating latency	1wU	0.475	0.228	2.080	0.037
	5wU	0.589	0.248	2.380	0.018
	1wF	0.176	0.181	0.970	0.333
	5wF	-0.415	0.253	-1.640	0.101
Proportion alive	1wU	-1.149E-15	3.890e-05	0	1
	5wU	0.993	0.149	6.650	0.095
	1wF	-0.697	1.065	-0.655	0.631
	5wF	-0.551	0.090	-6.107	0.103
Offspring number	1wU	-0.030	0.040	-0.740	0.460
	5wU	-0.079	0.064	-1.238	0.218
	1wF	-0.001	0.045	-0.011	0.991
	5wF	-0.286	0.160	-1.785	0.081
Paternity share	1wU	0.529	0.457	1.158	0.252
	5wU	0.201	0.368	0.546	0.586
	1wF	-0.112	0.294	-0.381	0.704
	5wF	-2.660	1.151	-2.312	0.023
Mating latency	1wU	-0.388	0.153	-2.540	0.011
	5wU	0.224	0.136	1.640	0.101
	1wF	0.042	0.150	0.280	0.778
	5wF	0.005	0.173	0.030	0.976
